# The role of magnetic sphincter augmentation in the treatment of gastroesophageal reflux disease

**DOI:** 10.1097/MOG.0000000000000748

**Published:** 2021-04-20

**Authors:** Luigi Bonavina, Nick Boyle, Sebastian F. Schoppmann

**Affiliations:** a University of Milan, Department of Biomedical Sciences for Health, Division of General and Foregut Surgery, IRCCS Policlinico San Donato, Milan, Italy; b King Edward VII Hospital, Medical Director RefluxUK, London, UK; c Medical University of Vienna, Department of General Surgery, Upper-GI-Service, Wien, Austria

**Keywords:** fundoplication, gastroesophageal reflux disease, lower esophageal sphincter, magnetic sphincter augmentation

## Abstract

Magnetic sphincter augmentation (MSA) has been designed as a less disruptive and more standardized laparoscopic surgical procedure than fundoplication for patients with early stage gastroesophageal reflux disease (GERD). We analyzed the more recent literature in search of updates regarding indications, technique, perioperative management, and long-term outcomes.

**Recent findings:**

Over the years, the procedure of MSA has evolved to including full hiatus repair rather than relying on the preservation of the phreno-esophageal ligament. Restoring the mechanical synergy between the lower esophageal sphincter and the crural diaphragm has the potential to further enhance the antireflux barrier. The adoption of this approach has led to expand the indications from early stage disease to different scenarios including patients with high esophageal acid exposure, atypical symptoms, large hiatal hernias, Barrett's esophagus, postbariatric surgery, and previously failed fundoplication.

**Summary:**

MSA has a favorable side-effect profile and is highly effective in reducing typical reflux symptoms, medication dependency, and esophageal acid exposure. Excellent outcomes have been confirmed over a 12-year follow-up, indicating that the operation has the potential to prevent GERD progression. Further studies are needed to confirm the cost-effectiveness of this procedure in patients with more advanced disease-stage and prior gastric surgery. A randomized control trial comparing MSA with fundoplication could raise the level of evidence and the strength of recommendation.

## INTRODUCTION

From the perspective of many gastroenterologists and patients, the treatment of gastroesophageal reflux disease (GERD) remains unsatisfactory. Although life-changing for some, proton-pump inhibitor (PPI) therapy frequently fails to control the symptomatic disease. More than one-third of patients are resistant or only partial responders to PPI therapy [1,2], and even escalation to high dose may be inadequate to maintain a symptom-free state and improve quality of life. Furthermore, many patients suffer from persistent nonacid reflux and nocturnal acid breakthrough that may lead to serious complications of the disease, such as volume regurgitation, pulmonary aspiration, Barrett's metaplasia, and esophageal adenocarcinoma.

Currently, laparoscopic Nissen fundoplication is considered to represent the reference gold standard. However, while safe and effective in specialist centers [3], recent evidence suggests that over 12% of operations are associated with complications, nearly 10% require revisional surgery, and clinical outcomes are dependent upon both surgeon expertise and center volume [4–6]. Among patients, fundoplication remains unpopular due to the perception of significant side effects including bloating and flatulence, which were reported to occur in 40% and 57% of patients, respectively in a multicenter European trial [7]. All these factors have impacted referral patterns, leaving a significant gap between the volume of potential surgical candidates and the real-world utilization of antireflux surgery. Indeed, it has been estimated that only 0.1% of GERD patients undergo antireflux surgery [8]. Consequently, many patients are left in the unsatisfactory position of enduring a life-time of medication dependence despite incomplete symptom relief, or undergoing a surgical procedure that alters gastric anatomy and has potential troublesome side-effects.

Magnetic sphincter augmentation (MSA) was developed as a less invasive and more reproducible surgical technique that would avoid some of the complications and side effects of fundoplication, and be more appealing to patients while potentially preventing disease progression and avoiding long-life pharmacologic therapy [9,10].

MSA using the Linx Reflux Management System (Ethicon, USA), approved by the FDA in 2012, was designed to be a fundic-sparing laparoscopic antireflux procedure. In this report, we review the latest advances with MSA in the management of GERD patients. 

**Box 1:**
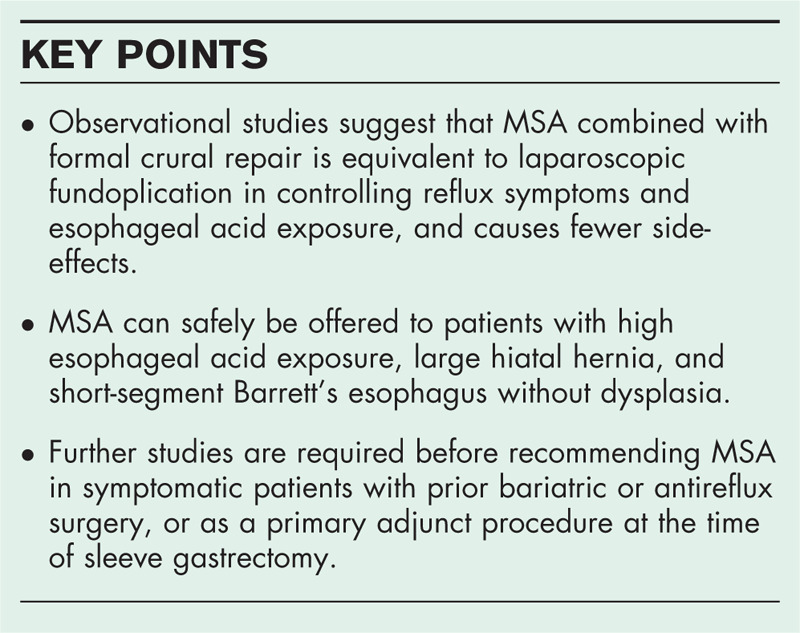
no caption available

## TECHNOLOGY AND RATIONALE OF MAGNETIC SPHINCTER AUGMENTATION

The MSA device is a mechanical tool designed to augment the physiologic lower esophageal sphincter (LES) barrier to reflux by magnetic force. It consists of a series of biocompatible titanium beads encasing magnetic cores. The beads are interlinked with independent titanium wires to form a flexible and expandable ring. At rest, each bead is in contact with adjacent beads. These beads can move independently, creating a dynamic implant with a ‘roman arch’ configuration that does not compress the esophagus or limit its range of motion upon swallowing, belching, and vomiting. The MSA device is manufactured in different sizes and is capable of nearly doubling its diameter in response to a swallowed bolus. Although augmenting the LES, the intrinsic design of the device permits expansion to accommodate a swallowed bolus; in the resting position, the magnetic bonds of the device provide resistance to opening of the LES when challenges of intragastric pressure elevations occur. After surgical implantation, the device becomes encased in a fibrous capsule that facilitates removal without damaging the esophageal wall. The MSA device has recently received magnetic resonance imaging approval for scanning in systems upto 1.5 T.

## SURGICAL TECHNIQUE

Compared to fundoplication, the current surgical standard, the MSA procedure requires less dissection and more standardized technical steps. The device is implanted laparoscopically. The first step is division of the peritoneum on the anterior surface of the gastroesophageal junction. The lateral surface of the left crus is freed from the posterior fundic wall without dividing any short gastric vessels. Although preserving the hepatic branch of the anterior vagus nerve, the gastro-hepatic ligament is opened to access the retro-esophageal window and to identify the posterior vagal nerve bundle. A tunnel is created between the vagus and the posterior esophageal wall, and the circumference of the esophagus is measured using a sizing tool to determine the appropriate size of the MSA, and allowing tailoring to each individual. The appropriate device is placed around the gastroesophageal junction between the esophageal wall and the posterior vagus nerve and is locked anteriorly. Depending on hernia size and surgeon's judgment, concurrent full mediastinal dissection and posterior crural repair can be performed. Operative time is typically less than one hour. Patients are discharged the same day of surgery or on the first postoperative day and are counseled to return to a regular diet and to discontinue PPI use.

## FEASIBILITY STUDIES AND SHORT- TO INTERMEDIATE-TERM OUTCOMES

Since the first human implantation in 2007, feasibility studies investigating the clinical outcomes of MSA have confirmed high rates of symptom relief, discontinuation of PPI therapy, objective reduction of esophageal acid exposure, and improved quality of life [11,12]. The primary inclusion criteria in the feasibility trial were age > 18 < 85 years, typical reflux symptoms at least partially responsive to PPI therapy, abnormal esophageal acid exposure, and normal contraction amplitude and waveform in the esophageal body. Patients with a history of dysphagia, previous upper gastrointestinal surgery, previous endoluminal antireflux procedures, sliding hiatal hernia > 3 cm, esophagitis > grade A, histologically proven Barrett's esophagus, and abnormal manometric findings (distal esophageal contraction amplitude less than 35 mmHg on wet swallows or < 70% propulsive peristaltic sequences) were excluded. Thirty-three patients were followed at 5 years. The mean total GERD-Health Related Quality of Life (HRQL) score off PPI decreased from 25.7 to 2.9 (*P* < 0.001), and 94% of patients had a greater than 50% reduction in the total score. pH normalization was achieved in 70% of patients. Complete PPI cessation or at least 50% reduction of the daily dose was achieved by 88% and 94% of patients, respectively. Overall, 43% of patients complained of mild dysphagia that resolved by 3 months without treatment. Three patients were explanted because of persistent dysphagia, persistent reflux, or the need to undergo magnetic resonance imaging. Similar rigorous inclusion criteria were applied in a large US multicenter study involving 100 patients [13]. Significant improvements were seen in GERD-related quality of life, regurgitation, and esophageal acid exposure. The use of PPI dropped to 13% at 3 years, and patient satisfaction with reflux control increased to 94% with most patients retaining the ability to belch and vomit. Dysphagia occurred in 68% of patients, but decreased to 4% by 3 years. Five percentage of patients rated the dysphagia as severe and the device was removed in 3 of them with complete symptom resolution. At 5-year follow-up, 75% of patients remained PPI free [14].

A recent randomized clinical trial compared MSA with high dose PPI at 1-year follow-up in patients with moderate to severe regurgitation. Non-responders to PPI were allowed to cross over to surgery. MSA resulted in control of regurgitation in 96% of patients and pH normalization in 70%. In comparison, only 19% of patients receiving PPI reported control of regurgitation [15^▪▪^].

## SAFETY PROFILE

Concerns regarding the safety of MSA, especially the fear of erosions, stem from past adverse events with the Angelchick prosthesis (a C-shaped silicon ring, considerably larger than the Linx device, that was fitted around the gastroesophageal junction and resulted in frequent complications including migration and erosion) and, more recently, with the gastric banding device. A recent analysis of the safety profile of nearly 10,000 MSA implants found 29 reported cases of erosion, with a median time at the presentation of 26 months and an erosion risk of 0.3% after 4 years. The major risk factor appeared to be a mismatch between device size and esophageal circumference, and smaller devices (size 12 and 13) were associated with higher rates of erosion. Reversal to normal anatomy after MSA removal has been consistently safe. Most patients underwent a 2-stage procedure with endoscopic removal of the eroded portion followed by delayed laparoscopic removal of the remaining beads [16]. One-stage laparoscopic removal and concurrent fundoplication have proven safe and effective as well, both in elective and emergency procedures [17].

## COMPARISON OF MAGNETIC SPHINCTER AUGMENTATION WITH FUNDOPLICATION

A recent meta-analysis of 19 observational studies and 12,697 patients showed that, compared with fundoplication, MSA conferred equivalent GERD control as measured by the requirement for postoperative PPI therapy and GERD specific-HRQL scores. MSA was associated with lesser gas bloat symptoms (OR 0.34, 95% CI 0.16–0.71) and greater ability to belch (OR 12.34, 95% CI 6.43–23.70). Reoperation was necessary in 3.3% of patients [18^▪^].

Another study analyzed the outcomes of MSA and laparoscopic fundoplication in a European, multicenter observational registry including 631 patients. Effectiveness and safety were similar with the two procedures, but MSA allowed a higher percentage of patients the ability to vomit as needed (97.6% vs 68%) at 3 years [19].

## INDEPENDENT PREDICTORS OF SUCCESS

Ayazi *et al.*[20^▪▪^] analyzed the outcomes of the largest single-institution series of 553 patients treated with MSA over a 5-year period. Favorable outcome was defined as freedom from PPI and > 50% improvement in GERD-HRQL score. Independent predictors of a favorable outcome were age < 45 years (OR 4.2, 95% CI 1.1–15.2), male sex (OR 2.5, 95% CI 1.1–5.7), GERD-HRQL total score > 15 (OR 7.5, CI 3.3–16.8), and an abnormal DeMeester score (OR 2.6, 95% CI 1.1–5.7).

## EFFECT OF MAGNETIC SPHINCTER AUGMENTATION ACROSS THE SPECTRUM OF DISEASE SEVERITY

Dunn *et al.*[21^▪^] reported a significant decrease in the length of intestinal metaplasia and no progression to dysplasia or adenocarcinoma in a series of 74 MSA patients with a preoperatively confirmed diagnosis of Barrett's esophagus. It should be noted, however, that the mean follow-up time of the entire cohort was 2.3 years. Another study [22^▪^] reported on 79 patients undergoing MSA for large (>3 cm) hiatal hernia. Over a median follow-up of 2.9 years, median GERD-HRQL scores, DeMeester scores, and severity of esophagitis decreased significantly, and the hiatal hernia recurrence rate was 6.3%.

Schwameis *et al.*[23^▪▪^], using a DeMeester score ≥50 as a cutoff point to define severe GERD, divided 334 MSA patients into two groups (DeMeester score ≥50 vs DeMeester score < 50) and compared patient characteristics and outcomes. At a mean postoperative follow-up of 13.6 months, GERD-HRQL scores, DeMeester scores, and prevalence of esophagitis were similar in the two groups. However, fewer patients with severe disease were likely to be free of PPI (85% vs 93.1%, *P* = 0.041). The rates of postoperative dysphagia and the need for device explant were similar in the two groups. The authors speculated that the circumferential arrangement of MSA provides superior antireflux control compared to Toupet fundoplication while limiting the side-effects of the Nissen.

## EFFECT OF MAGNETIC SPHINCTER AUGMENTATION ON PREDOMINANTLY ATYPICAL SYMPTOMS

Ward *et al.*[24^▪^] focused on the clinical outcomes of 86 patients with GERD who suffered primarily from atypical symptoms (chronic cough, hoarseness, throat clearing, and globus) resulting from laryngopharyngeal reflux. All these patients had an abnormal DeMeester score off PPI. Symptoms were assessed at baseline and 1-year postoperatively using GERD-HRQL and Reflux Symptom Index questionnaires. Both atypical and typical symptoms significantly improved one year after MSA implantation.

## DYSPHAGIA AND ESOPHAGEAL MOTILITY

Eligibility criteria for MSA use from the American College of Gastroenterology and the device manufacturer have so far recommended that preoperative dysphagia and abnormalities of esophageal motility seen on high-resolution manometry should exclude patients from MSA [25]. However, there is mixed evidence regarding whether preoperative manometry can predict outcomes. A recent study [26] using high-resolution manometry in a cohort of 45 patients found that preoperative dysphagia was the only factor significantly associated with postoperative dysphagia. Interestingly, ineffective esophageal motility reverted to normal after MSA in more than one-third of patients. Leeds *et al.*[27^▪^] reported that the value of preoperative distal contractile integral was lower, without reaching statistical significance, in patients who required endoscopic or surgical treatment. Ayazi *et al.*[28^▪^] reported that that less than 80% peristaltic contractions in the distal esophagus was a predictor of persistent dysphagia. Dominguez-Profeta *et al.*[29^▪^] found that using multiple rapid swallows during high-resolution manometry is useful to predict patients at risk for postoperative dysphagia. The esophago-gastric junction outflow resistance imposed by MSA was investigated in a series of 43 patients free of dysphagia at 1 year after surgery. Outflow resistance was measured by the intrabolus pressure recorded 2 cm proximal to the LES. Intrabolus pressure significantly increased after surgery, regardless of the MSA device size, and its upper normal limit was 30 mmHg. There was also a significant correlation between intrabolus pressure and the percentage of incomplete bolus clearance [30^▪▪^].

## INTRA-OPERATIVE MANAGEMENT

Leeds *et al.*[31^▪^] sent a 12-question survey to 37 certified MSA proctors to obtain consensus on indications and technical aspects of the procedure. There was 79% agreement in favor of full mediastinal dissection and concurrent hiatoplasty to restore at least 3 cm of intra-abdominal esophagus. However, a variety of sizing criteria were adopted. Recent data suggest that patients with a small (12–14 beads) vs a larger MSA implant (15–17 beads) had a significantly higher rate of postoperative dysphagia (58.5% vs 30.0% *P* = 0.026) [29^▪^]. Consequently, most surgeons now over-size by 3 (rather than 2) clicks of the sizing device.

## PROPHYLAXIS AND MANAGEMENT OF POST-OPERATIVE DYSPHAGIA

Short-term dysphagia has been reported in 43–83% of patients after MSA [32–34]. It appears that persistent postoperative dysphagia following MSA can be related to factors other than esophageal motility, such as sizing technique or excessive fibrosis, and that steroid pulses combined with 1 or 2 pneumatic dilation are effective in up to 75% of patients [35]. The results of a multicenter study showed that a through-the-scope 15 mm balloon dilator was 83.3% effective in relieving dysphagia for any size MSA implant, and the rate of MSA explant for persistent dysphagia was 12% [36^▪^].

Changes in device sizing protocols and patient management pathways have been recommended to reduce the need for dilatation. Increase in size of the device, frequent bites of solid food, use of oral steroids, and avoidance of early dilatations seem to have decreased the rate of postoperative dysphagia [28^▪^,29^▪^,^30^^▪▪^,31^▪^,^32^–^35^,36^▪^].

## LONG-TERM FOLLOW-UP DATA

Recently, Ferrari *et al.*[37^▪^] provided 6–12 year outcome data after MSA implant at a single institution. One-hundred-twenty-four patients had a minimum 6 years of follow-up and a median follow-up of 9 years. The mean GERD-HRQL score decreased from 19.9 at baseline to 4.01 at the latest office visit (*P* < 0.001). The prevalence of grade 2–4 regurgitation significantly decreased from 59.6% to 9.6%, and 79% of patients discontinued PPI use. Esophageal pH testing showed that the mean percentage time pH < 4 decreased from 9.7 to 4.2%, *P* < 0.001). Four patients who had been treated with radiofrequency ablation for short-segment Barrett's esophagus without dysplasia before the MSA procedure were followed up to 8 years without recurrence of intestinal metaplasia. Postoperative esophageal acid exposure was within normal limits in all these individuals. Independent baseline predictors of a favorable outcome were age at intervention < 40 years (OR 4.61, CI 1.29–16.45) and total GERD-HRQL score > 15 (OR 4.19, CI 1.39–12.63). There were three MSA removals and no erosions in this series.

## EFFECT OF MAGNETIC SPHINCTER AUGMENTATION ON PERSISTENT OR DE NOVO GERD AFTER SLEEVE GASTRECTOMY OR PRIOR FAILED ANTIREFLUX SURGERY

A systematic literature review found 7 studies and a total of 35 patients who underwent MSA implant mainly after sleeve gastrectomy or Roux-en-Y gastric bypass [38]. The results showed that MSA is feasible and safe after bariatric surgery, and the GERD-HRQL score significantly improved compared to baseline. A recent study by Leeds *et al.*[39^▪^] evaluated the outcomes of MSA in 21 patients with prior bariatric and antireflux surgery. Postoperative GERD-HRQL scores were similar to surgery-naïve patients, but there was an increased operative time and length of stay, and larger devices were required in patients with prior gastric surgery.

Finally, a propensity-matched analysis of the Metabolic and Bariatric Surgery Accreditation and Quality Improvement Program compared MSA performed at the time of sleeve gastrectomy or Roux-en-Y gastric bypass vs no MSA. Of note, the rate of preoperative GERD was similar in both patient groups. MSA did not increase complications at 30-days [40^▪^].

## CONCLUSIONS

MSA was developed as a less disruptive and more standardized laparoscopic option for symptomatic patients with uncomplicated GERD. The initial target population consisted mainly of patients who did not have severe GERD warranting conventional fundoplication. Long-term studies confirm that MSA is durable and highly effective in decreasing symptoms, PPI dependence, and esophageal acid exposure. The side-effect profile is minimal, and device erosions have been rare and not associated with mortality or long-term morbidity.

With experience, the indications for MSA have been expanded to include patients with high esophageal acid exposure, atypical symptoms, large hiatal hernia, Barrett's esophagus, and postsleeve gastrectomy. Randomized trials comparing MSA and fundoplication are needed to establish at which level of disease severity magnetic augmentation is sufficient to restore antireflux competency of the LES. Further research is needed before recommending MSA as a remedial procedure after bariatric surgery or prior antireflux surgery, or as a primary adjunct to sleeve gastrectomy.

## Acknowledgements

*We would like to thank AIRES (Associazione Italiana Ricerca Esofago) for assistance with the study.*

### Financial support and sponsorship

*There was no funding received for this work.*

### Conflicts of interest

*L.B.* received honoraria from Ethicon, Inc. as a consultant and proctor for the Linx procedure.

*N.B.* received honoraria from Ethicon, Inc. as a consultant and proctor for the Linx procedure.

*S.S.* received honoraria from Ethicon, Inc. as a consultant and proctor for the Linx procedure.

## REFERENCES AND RECOMMENDED READING

Papers of particular interest, published within the annual period of review, have been highlighted as:

▪ of special interest
▪▪ of outstanding interest
